# Ultrasound-Guided Posteromedial Fascial–Periarticular Injection for Mild-to-Moderate Knee Osteoarthritis: Twelve-Month WOMAC Outcomes in a Retrospective Pilot Case Series

**DOI:** 10.3390/diagnostics16152382

**Published:** 2026-07-29

**Authors:** King Hei Stanley Lam, Yonghyun Yoon, Teinny Suryadi, Daniel Chiung-Jui Su, Wei-Chuan Wang, Wai Wah Lai, Yuanita Ida, Kentaro Onishi

**Affiliations:** 1 Faculty of Medicine, The Chinese University of Hong Kong, New Territories, Hong Kong, China; 2 Faculty of Medicine, The University of Hong Kong, Hong Kong, China; 3 The Board of Clinical Research, The Hong Kong Institute of Musculoskeletal Medicine, Kowloon, Hong Kong, China; laiwwm@gmail.com; 4 The Board of Clinical Research, The International Association of Musculoskeletal Medicine, Kowloon, Hong Kong, China; painfreedoc22@gmail.com (T.S.); drdaniel@gmail.com (D.C.-J.S.); myoneme@gmail.com (W.-C.W.); 5 Department of Orthopaedics, International Academy of Regenerative Medicine, Incheon 21574, Republic of Korea; 6 Department of Orthopedic Surgery, Incheon Terminal Orthopedic Surgery Clinic, Incheon 21574, Republic of Korea; 7 Department of Orthopedic Surgery, Hallym University Kangnam Sacred Heart Hospital, Seoul 06143, Republic of Korea; 8 Department of Orthopaedics, MSKUS, San Diego, CA 92084, USA; 9 Department of Physical Medicine and Rehabilitation, Medistra Hospital, Jakarta 12950, Indonesia; 10 Department of Physical Medicine and Rehabilitation, Synergy Clinic, Jakarta 11510, Indonesia; 11 Department of Physical Medicine and Rehabilitation, Hermina Podomoro Hospital, Jakarta 14350, Indonesia; 12 Department of Physical Medicine and Rehabilitation, Chi Mei Medical Center, Tainan 710, Taiwan; 13 A Tempo Regeneration Center for Musicians, Tainan 700, Taiwan; 14 Department of Physical Medicine and Rehabilitation, Ultra Clinic, Taipei City 100, Taiwan; 15 Department of Physical Medicine and Rehabilitation, Mitra Keluarga Kenjeran Hospital, Surabaya 60114, Indonesia; idayuanita7@gmail.com; 16 Department of Physical Medicine and Rehabilitation, Mayapada Hospital, Surabaya 60256, Indonesia; 17 Department of Physical Medicine and Rehabilitation, RegenCore Method Clinic, San Francisco, CA 94115, USA; kenonishi918@gmail.com

**Keywords:** knee osteoarthritis, ultrasound-guided injection, WOMAC, dextrose, hydrodissection, posteromedial knee, retrospective study, musculoskeletal ultrasound, multimodal assessment

## Abstract

Knee osteoarthritis (OA) is increasingly recognized as a whole-joint disorder involving periarticular soft tissues in addition to intra-articular structures. A recently published technical report described an ultrasound-guided posteromedial knee injection targeting a fascial–periarticular convergence region operationally termed the popliteal fascial retinaculum. In the present study, we evaluated preliminary longitudinal clinical outcomes after this procedure in patients with mild-to-moderate knee OA. **Background/Objectives:** Knee OA is a common cause of chronic pain, stiffness, and disability. In the present study, we evaluated preliminary 12-month WOMAC outcomes after a three-session ultrasound-guided posteromedial fascial–periarticular injection in patients with Kellgren–Lawrence grade 1–3 knee OA. **Methods:** This retrospective analysis of a prospectively maintained single-center clinical registry included 10 patients treated between October 2023 and October 2025. Each patient received three monthly ultrasound-guided posteromedial injections using 30 mL of 5% dextrose with 0.1% lidocaine (lignocaine). The Western Ontario and McMaster Universities Osteoarthritis Index (WOMAC) was recorded at baseline and at 1, 3, 6, and 12 months after the final injection. Data are mean ± SD, median (IQR), and 95% CI. In exploratory inferential analyses, we used Friedman tests and Wilcoxon signed-rank tests with Bonferroni correction. **Results:** The mean WOMAC total score improved from 59.5 ± 4.33 at baseline to 38.6 ± 4.50 at 1 month, 34.3 ± 4.45 at 3 months, 30.6 ± 3.06 at 6 months, and 28.5 ± 2.51 at 12 months after the final injection, corresponding to a mean 12-month within-patient change of −31.0 points (95% CI −32.58 to −29.42; −52.1%). Mean pain improved from 12.6 ± 1.35 to 5.6 ± 0.70, stiffness from 4.7 ± 0.48 to 2.1 ± 0.32, and physical function from 42.2 ± 2.62 to 20.8 ± 2.15. All 10 patients had lower WOMAC total scores at 12 months than at baseline. Friedman tests were significant for all four WOMAC domains (all *p* ≤ 5.05 × 10^−7^); Wilcoxon signed-rank tests versus baseline reached the theoretical minimum *p* at *n* = 10 (Bonferroni-adjusted *p* = 7.81 × 10^−3^) at every follow-up timepoint. No serious adverse events were documented; mild transient post-procedural soreness occurred in three patients. **Conclusions:** In this small retrospective pilot cohort, ultrasound-guided posteromedial fascial–periarticular injection was associated with sustained improvement in WOMAC pain, stiffness, physical function, and total scores over 12 months. These findings are preliminary and hypothesis-generating; non-specific effects including placebo, regression to the mean, and natural symptomatic fluctuation cannot be excluded. Prospective controlled trials are required to establish safety, reproducibility, mechanism, and comparative effectiveness.

## 1. Introduction

Knee osteoarthritis (OA) is a common cause of chronic pain, stiffness, and mobility limitation and is a major contributor to disability worldwide [1]. Although OA was historically conceptualized primarily as a disorder of articular cartilage, contemporary understanding increasingly recognizes it as a whole-joint disease involving the synovium, capsule, ligaments, tendon insertions, menisci, subchondral bone, and adjacent periarticular soft tissues [1,2]. This broader framework has contributed to growing interest in image-guided interventions that address extra-articular and periarticular pain-generating tissues in addition to intra-articular pathology.

Current clinical practice guidelines recommend multimodal management centered on education, exercise, weight management when appropriate, and selective use of pharmacologic and procedural interventions [3]. Nevertheless, many patients continue to experience persistent pain and functional limitation despite conservative treatment, sustaining ongoing interest in image-guided approaches that may target symptom-relevant periarticular tissues in selected patients [3,4].

Fascial tissues are increasingly recognized as biologically active connective tissues involved in force transmission, proprioception, and nociception [5,6]. In the posterior knee, the popliteal fascial complex exhibits multilayered continuity with hamstring-associated fascial structures proximally and crural fascia distally [7]. Such anatomical continuity raises the possibility that interfascial and periarticular dysfunction in the posteromedial knee may contribute to symptoms and functional impairment in selected patients with OA.

Musculoskeletal ultrasound has become an increasingly useful adjunct in the evaluation and treatment of knee OA. Ultrasound can depict osteophytes, synovial changes, effusion, and periarticular soft-tissue abnormalities in real time, with acceptable validity, reliability, and correlation to MRI in several contexts [4,8]. Correlations between ultrasound-detected features and pain severity have been reported, supporting the use of ultrasound not only for diagnosis and phenotyping but also for accurate guidance of regional interventions [8]. In this way, ultrasound integrates diagnostic, phenotyping, and procedural roles within a single multimodal assessment framework.

Dextrose-based injections have been studied in knee OA in several clinical contexts, including prolotherapy-type approaches and hydrodissection-related applications [9,10,11,12]. Proposed mechanisms remain incompletely defined but may include effects on nociceptive signaling, local tissue environment, and mechanically constrained soft-tissue planes [13,14,15]. However, outcome data remain limited for posteromedial fascial–periarticular injection strategies specifically. A separate technical report recently described an ultrasound-guided posteromedial knee injection targeting a convergence region involving the semimembranosus insertion, popliteus-related structures, posterior capsule, oblique popliteal ligament, and proximal superficial medial collateral ligament, operationally termed the popliteal fascial retinaculum [16]. That publication focused on procedural description and cadaveric dye-distribution findings rather than patient outcomes.

The present study reports longitudinal clinical outcomes after that previously described technique in a retrospective pilot cohort of patients with Kellgren–Lawrence grade 1–3 knee OA [17]. The objective was to describe changes in WOMAC pain, stiffness, physical function, and total scores from baseline to 12 months after the final injection [18,19].

## 2. Materials and Methods

### 2.1. Study Design

This study is a retrospective analysis of a prospectively maintained single-center clinical registry of patients with symptomatic knee osteoarthritis treated with ultrasound-guided posteromedial fascial–periarticular injection at a musculoskeletal ultrasound-guided injection center in Hong Kong. Clinical registry data were collected between October 2023 and October 2025. Clinical assessments, including WOMAC, were performed as part of routine care at each visit; retrospective statistical analysis was performed after all included patients had completed the 12-month follow-up assessment.

### 2.2. Ethics

The study was conducted in accordance with the Declaration of Helsinki. Ethical oversight for the retrospective analysis of anonymized clinical registry data was provided by the Institutional Review Board of Chi Mei Medical Center, Taiwan (approval no. 11411-033; date of approval: 16 October 2025), under a pre-established collaborative research and data-sharing agreement between the treatment site and the reviewing institution. Only fully de-identified data were transferred for analysis; no directly identifiable patient information was transmitted across sites or across borders. Written informed consent for treatment and for the use of anonymized clinical data in publication was obtained from all patients.

### 2.3. Patients

During the registry period, 14 patients were screened for eligibility. Four patients were excluded (2 with inflammatory arthropathy identified on chart review, 1 with prior partial knee arthroplasty on the index side, and 1 who had not yet completed the 12-month follow-up window at the time of data lock). Ten consecutive eligible patients with complete 12-month follow-up were included.

Inclusion criteria were symptomatic unilateral knee OA with radiographic Kellgren–Lawrence grade 1–3 disease [17] and failure of at least 3 months of conservative management. Exclusion criteria were inflammatory arthritis, active infection, corticosteroid or biologic injection within 3 months, prior major knee surgery, and marked coronal deformity.

The source clinical records included age, sex, treated side, body mass index (BMI), Kellgren–Lawrence grade, symptom duration, prior conservative treatments, ongoing physiotherapy, oral analgesic/NSAID use, and any additional interventions during follow-up. Descriptive follow-up context variables are summarized in Supplementary Table S3.

### 2.4. Injection Protocol

Each patient underwent three monthly ultrasound-guided injections using 30 mL of 5% dextrose with 0.1% lidocaine (lignocaine). The detailed procedural technique has been published separately [16]. Briefly, the patient was positioned prone with slight knee flexion, posteromedial landmarks were identified under ultrasound guidance after safety mapping of the popliteal neurovascular structures, and injectate was distributed by hydrodissection across the intended posteromedial fascial–periarticular target planes. All procedures were performed by a single experienced operator.

**Figure 1:**
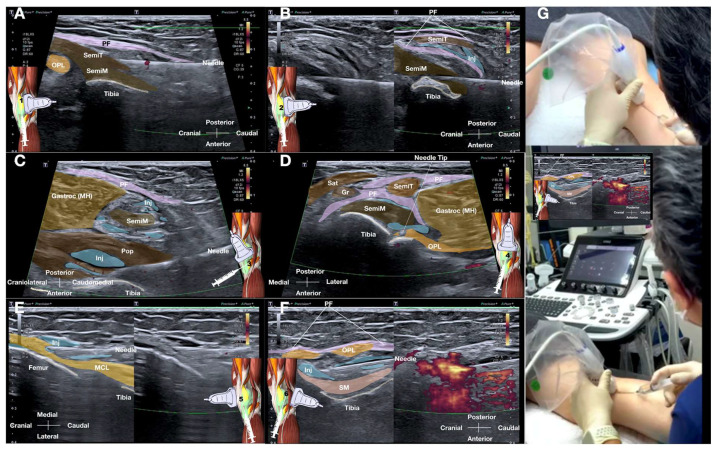
Representative still images illustrating the ultrasound-guided posteromedial injection approach used in this study. This figure presents selected still images from the procedural demonstration video, summarizing patient positioning (**G**), transducer placement, the in-plane needle trajectory, and the posteromedial fascial–periarticular target region used for injection. The principal target structures include the popliteal fascia (**A**), semimembranosus (**B**), popliteus tendon and muscle (**C**), oblique popliteal ligament (**D**), medial collateral ligament (**E**), and the fibrous layer and synovial membrane of the posterior knee articular capsule (**F**). The figure is provided for general procedural orientation only. The full dynamic procedural demonstration is available in Video S1. A detailed technical description and cadaveric feasibility study were reported separately [16]. *Abbreviations:* Gastroc (MH), medial head of gastrocnemius; Gr, gracilis; Inj, injectate; MCL, medial collateral ligament; OPL, oblique popliteal ligament; PF, popliteal fascia; POP, popliteus; Sat, sartorius; SemiM, semimembranosus; SemiT, semitendinosus; SM, synovial membrane.

Representative still images from the procedural demonstration are shown in Figure 1, and the full dynamic sequence of probe positioning, target identification, and needle advancement is provided in Video S1.

### 2.5. Outcome Measures

**Figure 2:**
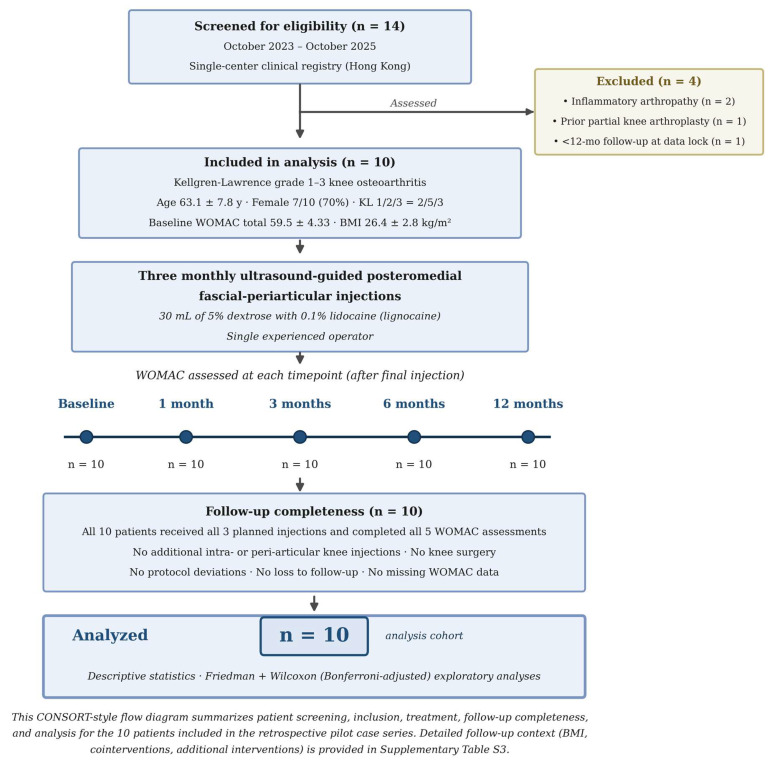
CONSORT-style patient flow and study timeline. Retrospective analysis of a prospectively maintained single-center clinical registry (October 2023–October 2025). Fourteen patients were screened for eligibility; four were excluded (2 with inflammatory arthropathy, 1 with prior partial knee arthroplasty on the index side, and 1 who had not yet completed the 12-month follow-up window at the time of data lock). Ten consecutive eligible patients with Kellgren–Lawrence grade 1–3 knee osteoarthritis were included in the analysis (mean age 63.1 ± 7.8 years; 7/10 female; KL grade 1/2/3 = 2/5/3; mean baseline WOMAC total 59.5 ± 4.33; mean BMI 26.4 ± 2.8 kg/m^2^). All 10 patients received three monthly ultrasound-guided posteromedial fascial–periarticular injections (30 mL of 5% dextrose with 0.1% lidocaine [lignocaine]) delivered by a single experienced operator, and completed WOMAC assessments at baseline and at 1, 3, 6, and 12 months after the final injection. The analysis cohort at each CONSORT stage is *n* = 10, as highlighted by the prominent “*n* = 10” badge at the Analyzed stage. No patient received an additional intra- or periarticular knee injection or underwent knee surgery during the 12-month follow-up; there were no protocol deviations, no loss to follow-up, and no missing WOMAC data. Detailed follow-up context (BMI change, cointerventions, and additional interventions) is provided in Supplementary Table S3.

The primary outcome measure was the Western Ontario and McMaster Universities Osteoarthritis Index (WOMAC), a validated patient-reported outcome instrument widely used in hip and knee OA [18,19]. WOMAC subscales included pain (0–20), stiffness (0–8), and physical function (0–68), with a total score of 0–96; higher scores indicate worse symptoms and disability. Assessments were performed at baseline before treatment and at 1, 3, 6, and 12 months after the final injection (Figure 2).

Secondary descriptive outcomes included treatment completion, adverse events, and patient global satisfaction at 12 months. Patient global satisfaction was recorded using a single-item 5-point Likert scale (1 = very dissatisfied; 5 = very satisfied) administered as part of routine clinical follow-up. This item is not a formally validated instrument and is reported here only as a descriptive secondary outcome, consistent with common practice in prior case series.

### 2.6. Statistical Analysis

Given the small sample size and exploratory retrospective design, the primary analysis was descriptive. Continuous variables are summarized as mean ± standard deviation (SD), median (interquartile range, IQR), and 95% confidence intervals (CIs) at each timepoint. Absolute and percentage changes from baseline in WOMAC total score, with 95% CIs for the paired within-patient change, are reported at each follow-up interval.

As an exploratory inferential analysis, a Friedman test was applied to each WOMAC domain across the 5 timepoints. Pairwise post hoc comparisons between each follow-up timepoint and baseline were performed using the Wilcoxon signed-rank test with Bonferroni correction for four comparisons per domain. Paired t-tests and Cohen’s dz were computed as sensitivity analyses. All inferential results are considered exploratory and hypothesis-generating; no confirmatory causal inference is intended. Two-sided *p* < 0.05 (Bonferroni-adjusted where indicated) was regarded as nominally significant. Analyses were performed in Python 3.11.9 using SciPy 1.14.1.

As this was a pilot case series intended to describe the preliminary longitudinal signal of a novel ultrasound-guided posteromedial approach that had previously been reported only as a technical and cadaveric study, no formal a priori sample size calculation was performed; the enrolled cohort represents all consecutive eligible patients with complete 12-month follow-up within the registry period, and effect estimates are reported with 95% confidence intervals to support the design of an adequately powered prospective controlled trial.

## 3. Results

### 3.1. Patient Cohort

Ten patients were included in the analysis. All patients completed baseline and follow-up WOMAC assessments through 12 months after the final injection. No missing WOMAC data were present in the analyzed dataset.

The cohort had a mean age of 63.1 ± 7.8 years, included seven women, had a mean BMI of 26.4 ± 2.8 kg/m^2^, and had a Kellgren–Lawrence distribution of grade 1 in two patients, grade 2 in five patients, and grade 3 in three patients (Table 1).

During the 12-month follow-up, no patient received an additional intra- or periarticular knee injection, and no patient underwent knee surgery. Mean BMI was 26.4 ± 2.8 kg/m^2^ at baseline and did not change by more than 0.5 kg/m^2^ at 12 months. Ongoing physiotherapy and analgesic/NSAID use during follow-up are summarized in Supplementary Table S3.

### 3.2. WOMAC Pain

**Figure 3:**
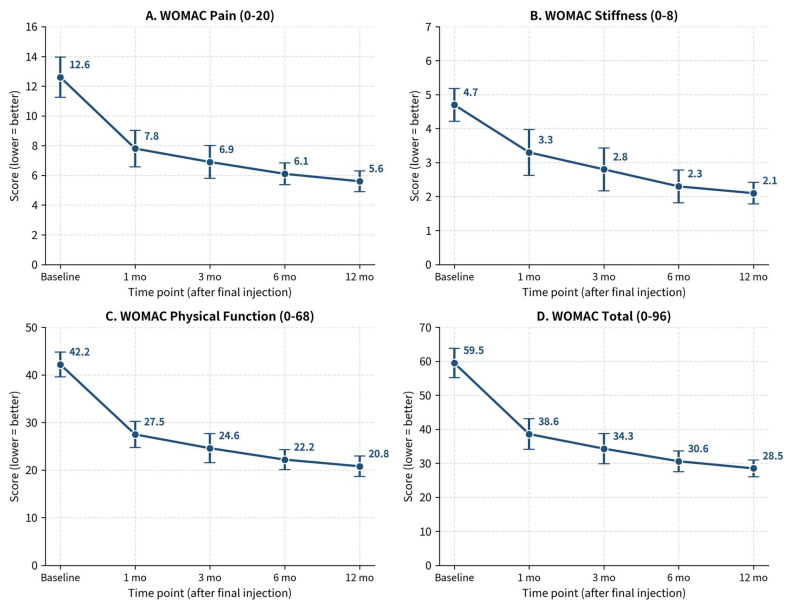
Mean WOMAC pain (**A**), stiffness (**B**), physical function (**C**), and total score (**D**) from baseline to 12 months after the final injection (*n* = 10). Data are mean ± SD. Lower scores indicate improvement.

The mean WOMAC pain score decreased from 12.6 ± 1.35 at baseline to 7.8 ± 1.23 at 1 month, 6.9 ± 1.10 at 3 months, 6.1 ± 0.74 at 6 months, and 5.6 ± 0.70 at 12 months after the final injection (Table 2; Figure 3A).

### 3.3. WOMAC Stiffness

The mean WOMAC stiffness score decreased from 4.7 ± 0.48 at baseline to 3.3 ± 0.67 at 1 month, 2.8 ± 0.63 at 3 months, 2.3 ± 0.48 at 6 months, and 2.1 ± 0.32 at 12 months (Table 2; Figure 3B).

### 3.4. WOMAC Physical Function

The mean WOMAC physical function score decreased from 42.2 ± 2.62 at baseline to 27.5 ± 2.76 at 1 month, 24.6 ± 3.06 at 3 months, 22.2 ± 2.10 at 6 months, and 20.8 ± 2.15 at 12 months (Table 2; Figure 3C).

### 3.5. WOMAC Total

**Figure 4:**
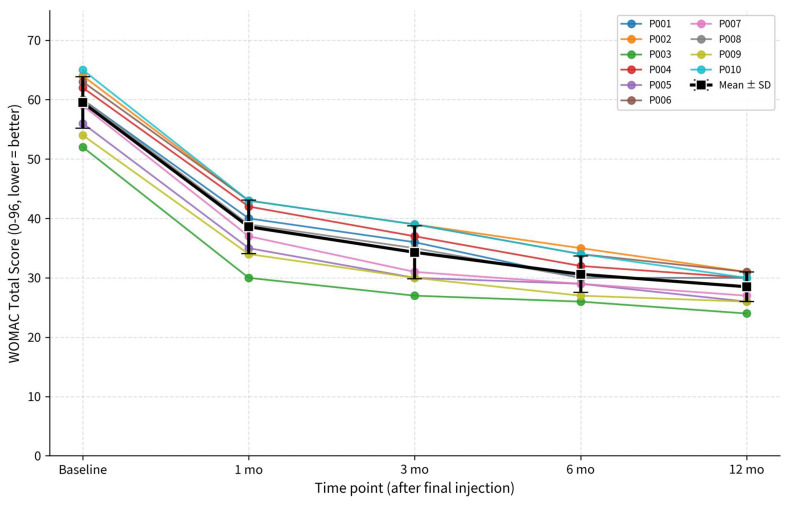
Individual patient trajectories for WOMAC total score from baseline to 12 months after the final injection (*n* = 10). Colored lines represent individual patients; the black line represents mean ± SD. All 10 patients had lower WOMAC total scores at 12 months than at baseline.

The mean WOMAC total score improved from 59.5 ± 4.33 at baseline to 38.6 ± 4.50 at 1 month, 34.3 ± 4.45 at 3 months, 30.6 ± 3.06 at 6 months, and 28.5 ± 2.51 at 12 months after the final injection. The mean absolute within-patient improvement from baseline was 20.9 points at 1 month (95% CI 20.27–21.53), 25.2 points at 3 months (95% CI 24.32–26.08), 28.9 points at 6 months (95% CI 27.71–30.09), and 31.0 points at 12 months (95% CI 29.42–32.58), corresponding to a 52.1% reduction relative to baseline (Table 2 and Table 3; Figure 3D and Figure 4). The individual patient WOMAC domain and total scores at all timepoints are provided in Supplementary Table S1.

### 3.6. Longitudinal Pattern

Improvement was evident at the first post-treatment assessment and remained present throughout follow-up after the final injection. All 10 patients had lower WOMAC total scores at 12 months after the final injection than at baseline. Individual patient trajectories showed a consistent overall downward trend in total WOMAC score across follow-up (Figure 4).

### 3.7. Safety and Patient Global Satisfaction

All patients completed the planned three monthly injections and the scheduled follow-up assessments through 12 months after the final injection. No serious adverse events were documented. Mild transient post-procedural soreness was reported in three patients and resolved spontaneously without intervention.

At 12 months after the final injection, patient global satisfaction was favorable, with eight patients (80%) reporting that they were very satisfied and two patients (20%) reporting that they were satisfied. No patient reported a neutral, dissatisfied, or very dissatisfied rating.

### 3.8. Exploratory Inferential Analyses

Friedman tests across the five timepoints yielded χ^2^(4) = 37.97 (*p* = 1.14 × 10^−7^) for pain, 34.87 (*p* = 4.94 × 10^−7^) for stiffness, 38.45 (*p* = 9.04 × 10^−8^) for physical function, and 39.68 (*p* = 5.05 × 10^−8^) for total score. Pairwise Wilcoxon signed-rank tests between each follow-up timepoint and baseline yielded the minimum achievable *p* at *n* = 10 (raw *p* = 1.95 × 10^−3^; Bonferroni-adjusted *p* = 7.81 × 10^−3^) for every domain and every timepoint, because all within-patient changes were in the direction of improvement. These results are provided in Table 3 and Supplementary Table S2 and should be interpreted as exploratory only, given the small sample and single-arm design.

## 4. Discussion

In this retrospective pilot case series, we found that patients with mild-to-moderate knee OA who underwent a three-session series of ultrasound-guided posteromedial fascial–periarticular injections showed improvement in WOMAC pain, stiffness, physical function, and total scores over 12 months after the final injection. Improvement was apparent at 1 month after the final injection and persisted through the final follow-up timepoint.

The present study is intentionally distinct from the previously published technical report [16]. That publication established procedural feasibility and cadaveric dye distribution, whereas the current study addresses longitudinal patient-reported outcomes. Accordingly, the present findings should be interpreted as preliminary and hypothesis-generating rather than confirmatory evidence of efficacy.

The observed pattern of improvement is clinically interesting, particularly because functional improvement contributed substantially to the reduction in total WOMAC score. Symptoms and disability in knee OA are influenced not only by intra-articular structural degeneration but also by periarticular soft-tissue dysfunction, pain sensitization, altered loading, and movement adaptations [1,2,5,6,7]. A posteromedial interfascial–periarticular approach may therefore be relevant in selected patients, although the present data do not establish a specific therapeutic mechanism.

Several biologically plausible mechanisms remain possible, including hydrodissection-related separation of interfascial planes, regional fluid redistribution, and modulation of nociceptive input from periarticular tissues, all of which have been discussed in the prior dextrose-injection and hydrodissection literature [13,14,15]. The uncontrolled design of the present study does not allow differentiation among these possibilities.

**Alternative explanations for the observed improvement.** Because this study was uncontrolled and retrospective, the observed within-patient improvements cannot be attributed solely to the intervention. Several non-specific mechanisms should be considered. Placebo and expectancy effects are well documented in injection-based OA trials and can produce clinically meaningful WOMAC changes over 6–12 months. Regression to the mean is plausible because patients typically present for injection when symptoms are at a personal high point, and symptomatic knee OA fluctuates over time. Natural symptomatic fluctuation may also account for some component of the observed improvement, particularly in patients with Kellgren–Lawrence grade 1–2 disease in whom episodes of flare and quiescence are common. Contextual effects including intensive clinician–patient interaction, the perceived novelty of the ultrasound-guided procedure, and increased attention to activity modification may also contribute. The consistency of directional improvement across all 10 patients through 12 months does not by itself rule out these non-specific explanations, and we deliberately refrain from causal language.

**Clinical magnitude relative to published thresholds.** The 12-month mean WOMAC total change (−31.0 points on 0–96, corresponding to a −52.1% reduction from baseline) exceeds published minimal important change and minimal important difference values for knee OA outcome tools reported in the systematic review by Silva et al. [20], and exceeds meaningful within-patient change thresholds derived from large tanezumab datasets by Conaghan et al. [21], which correspond to a 1-category improvement on the Patient Global Assessment of OA of approximately 12.5–16.2% and a 2-category improvement of approximately 25.0–32.5%. All 10 patients in the present cohort exceeded even the 2-category meaningful within-patient change threshold at 12 months. The observed subscale improvements were also of similar magnitude: mean WOMAC pain decreased by 7.0 points (from 12.6 ± 1.35 to 5.6 ± 0.70; −55.6%) and mean WOMAC physical function decreased by 21.4 points (from 42.2 ± 2.62 to 20.8 ± 2.15; −50.7%), both of which exceed the 2-category meaningful within-patient change thresholds reported by Conaghan et al. [21] for the respective subscales. However, published thresholds were derived in different populations and different comparators, and their applicability to a small uncontrolled pilot cohort is limited.

Prior randomized and observational studies of intra-articular or periarticular dextrose injections in knee OA have generally reported clinically meaningful WOMAC improvements over 6–12 months, with pooled standardized mean differences favoring dextrose over exercise or saline controls in some but not all analyses [9,10,22,23]. A recent randomized trial by Teymouri et al. [24] reported improvement in both dextrose-prolotherapy and intra-articular normal saline groups without a statistically significant between-group difference at 8 weeks, underscoring the substantial non-specific effects of image-guided injection in this population. The present study cannot be directly compared with these trials because of design, injectate composition (30 mL of 5% dextrose with 0.1% lidocaine), and target region (posteromedial fascial–periarticular rather than strictly intra-articular). Our data should therefore be regarded as complementary and hypothesis-generating rather than confirmatory.

**Diagnostic and multimodal assessment context.** Musculoskeletal ultrasound is increasingly recognized as a cost-effective, real-time adjunct to radiography and MRI for the assessment of knee OA, with correlations between ultrasound-detected features (synovial thickening, effusion, osteophytes, and periarticular soft-tissue abnormalities) and pain severity [4,8,25]. In the present study, ultrasound served both as a diagnostic phenotyping tool at intake and as the procedural guidance modality. The integration of imaging phenotyping, patient-reported outcomes, and procedural targeting is consistent with contemporary multimodal-assessment frameworks for musculoskeletal conditions and aligns with the scope of this Special Issue.

**Limitations.** This study has several important limitations. (i) It is retrospective and uncontrolled, with no comparator arm. (ii) The sample size is small (*n* = 10) and provides limited statistical precision; all inferential analyses are exploratory. (iii) All procedures were performed by a single experienced operator at a single center, limiting external generalizability. (iv) Outcome assessment was not blinded, introducing potential observer and reporter bias. (v) Selection bias is possible, as patients were treated in routine care and consented to procedural intervention. (vi) Regression to the mean and natural fluctuation of knee OA symptoms may contribute to the observed improvement. (vii) Placebo, contextual, and expectancy effects cannot be excluded. (viii) The injectate combined 5% dextrose with 0.1% lidocaine; the independent contribution of each component cannot be determined. (ix) The clinical target should be understood as a regional posteromedial fascial–periarticular access approach rather than a strictly focal single-structure injection, based on prior cadaveric dye-distribution findings [16]. (x) BMI change, physiotherapy participation, and analgesic use during follow-up were recorded from routine clinical notes rather than a structured CRF and are reported descriptively only (Supplementary Table S3). (xi) Objective imaging or biomechanical correlates were not available. (xii) No formal safety powering was performed; absence of serious adverse events in 10 patients does not exclude rare adverse events. (xiii) The patient global satisfaction item is a single-item non-validated Likert scale and should be interpreted only as a descriptive supplement to WOMAC. These limitations reinforce that the present findings are preliminary and hypothesis-generating and must be validated in prospective controlled trials.

Despite these limitations, this study also has practical strengths. Follow-up extended to 12 months after the final injection, all patients had complete WOMAC data, individual raw data are provided in Supplementary Table S1, and the direction of change was consistent across the cohort. These preliminary data may help inform the design of future prospective studies.

Future research should include prospective controlled trials with predefined adverse-event monitoring, standardized cointervention control, blinded outcome assessment, multi-operator and multi-center recruitment, and formal sample size calculation. Comparative studies against sham procedures, exercise-based care, or established injection strategies would be particularly valuable. Mechanistic studies using imaging, elastography, or functional biomechanical measures may also help clarify the role of this intervention within a multimodal musculoskeletal assessment framework.

## 5. Conclusions

In this retrospective pilot cohort of 10 patients with Kellgren–Lawrence grade 1–3 knee osteoarthritis, a three-session series of ultrasound-guided posteromedial fascial–periarticular injections was associated with sustained improvement in WOMAC pain, stiffness, physical function, and total scores through 12 months after the final injection. These results are preliminary and hypothesis-generating, and cannot be attributed causally to the intervention because of the retrospective, uncontrolled, single-center, single-operator design and the possibility of placebo, regression to the mean, and natural symptomatic fluctuation. Prospective controlled trials are required to establish safety, reproducibility, mechanism, and comparative effectiveness.

## Figures and Tables

**Table 1:** Baseline characteristics of the study cohort.

Variable	Value
Number of patients	10
Age, mean ± SD, years	63.1 ± 7.8
Female sex, *n* (%)	7 (70%)
BMI, mean ± SD, kg/m^2^	26.4 ± 2.8
Treated side, right/left, *n*	6/4
Kellgren–Lawrence grade 1/2/3, *n*	2/5/3
Symptom duration, mean ± SD, months	28.5 ± 11.4
Prior physiotherapy, *n* (%)	10 (100%)
Prior oral analgesic use, *n* (%)	10 (100%)
Prior intra-articular injection, *n* (%)	6 (60%)

Abbreviations: BMI, body mass index; SD, standard deviation.

**Table 2:** WOMAC scores from baseline to 12 months after the final injection (*n* = 10).

Timepoint	Domain	Mean ± SD	Median (IQR)	95% CI
Baseline	Pain (0–20)	12.6 ± 1.35	13.0 (12.0–13.8)	11.63–13.57
Baseline	Stiffness (0–8)	4.7 ± 0.48	5.0 (4.2–5.0)	4.35–5.05
Baseline	Function (0–68)	42.2 ± 2.62	42.0 (40.5–44.0)	40.33–44.07
**Baseline**	**Total (0–96)**	**59.5 ± 4.33**	**60.0 (56.8–62.8)**	**56.40–62.60**
1 month	Pain	7.8 ± 1.23	8.0 (7.0–9.0)	6.92–8.68
1 month	Stiffness	3.3 ± 0.67	3.0 (3.0–4.0)	2.82–3.78
1 month	Function	27.5 ± 2.76	28.0 (25.5–30.0)	25.53–29.47
**1 month**	**Total**	**38.6 ± 4.50**	**39.5 (35.5–42.8)**	**35.38–41.82**
3 months	Pain	6.9 ± 1.10	7.0 (6.0–8.0)	6.11–7.69
3 months	Stiffness	2.8 ± 0.63	3.0 (2.2–3.0)	2.35–3.25
3 months	Function	24.6 ± 3.06	24.5 (22.2–27.8)	22.41–26.79
**3 months**	**Total**	**34.3 ± 4.45**	**35.5 (30.2–38.5)**	**31.12–37.48**
6 months	Pain	6.1 ± 0.74	6.0 (6.0–6.8)	5.57–6.63
6 months	Stiffness	2.3 ± 0.48	2.0 (2.0–2.8)	1.95–2.65
6 months	Function	22.2 ± 2.10	22.0 (21.0–23.8)	20.70–23.70
**6 months**	**Total**	**30.6 ± 3.06**	**30.0 (29.0–33.5)**	**28.41–32.79**
12 months	Pain	5.6 ± 0.70	5.5 (5.0–6.0)	5.10–6.10
12 months	Stiffness	2.1 ± 0.32	2.0 (2.0–2.0)	1.87–2.33
12 months	Function	20.8 ± 2.15	21.5 (19.0–22.8)	19.26–22.34
**12 months**	**Total**	**28.5 ± 2.51**	**30.0 (26.2–30.0)**	**26.71–30.29**

Data are mean ± SD, median (IQR), and 95% CI. IQR, interquartile range (25th–75th percentile). Lower scores indicate improvement.

**Table 3:** Change from baseline in WOMAC total score at each follow-up, with 95% CI and exploratory *p*-values (*n* = 10).

Timepoint	Mean Total ± SD	Mean Δ	95% CI of Δ	% Change	Wilcoxon *p* (Bonf.)	Paired t *p* (Bonf.)	Cohen’s dz
1 month	38.6 ± 4.50	−20.9	−21.53 to −20.27	−35.1%	0.0078	2.5 × 10^−13^	23.87
3 months	34.3 ± 4.45	−25.2	−26.08 to −24.32	−42.4%	0.0078	1.0 × 10^−12^	20.50
6 months	30.6 ± 3.06	−28.9	−30.09 to −27.71	−48.6%	0.0078	4.4 × 10^−12^	17.37
12 months	28.5 ± 2.51	−31.0	−32.58 to −29.42	−52.1%	0.0078	3.0 × 10^−11^	14.02

Δ, mean paired within-patient change (baseline−follow-up); a positive change indicates improvement. Wilcoxon signed-rank test used as the primary exploratory inferential test with Bonferroni correction for four comparisons; paired t-test shown as a sensitivity analysis. Friedman test across all five timepoints for WOMAC total: χ^2^(4) = 39.68, *p* = 5.05 × 10^−8^. Inferential *p*-values are exploratory and hypothesis-generating; the small sample size (*n* = 10) does not permit confirmatory causal inference. All Wilcoxon *p*-values reach the minimum achievable at *n* = 10 (1.95 × 10^−3^ raw; 7.81 × 10^−3^ after Bonferroni correction) because every within-patient change was in the same direction.

## Data Availability

The original contributions presented in this study are included in the article/Supplementary Materials. Further inquiries can be directed to the corresponding author.

## Supplementary Materials

The following supporting information can be downloaded at: https://www.mdpi.com/article/10.3390/diagnostics16152382/s1. Table S1: Individual raw WOMAC domain and total scores at baseline and follow-up. Table S2: Exploratory inferential analyses (Friedman and Wilcoxon signed-rank tests). Table S3: Descriptive follow-up context (BMI, cointerventions, and additional interventions). Video S1: Dynamic procedural demonstration of the ultrasound-guided posteromedial fascial–periarticular injection approach.
