# Supplementary material for: Ultrasound-Guided Posteromedial Fascial–Periarticular Injection for Mild-to-Moderate Knee Osteoarthritis: Twelve-Month WOMAC Outcomes in a Retrospective Pilot Case Series
Source: Diagnostics (Basel). 2026 Jul 29;16(15):2382. doi: 10.3390/diagnostics16152382 (PMC13464791; doi:10.3390/diagnostics16152382)
Supplement: Supplementary file 1 [file diagnostics-16-02382-s001.zip › diagnostics-4390108-supplementary.pdf]

Supplementary Materials

Ultrasound-Guided Posteromedial Fascial-Periarticular Injection for Mild-to-Moderate Knee Osteoarthritis: Twelve-Month WOMAC Outcomes in a Retrospective Pilot Case Series

King Hei Stanley Lam, Yonghyun Yoon, Teinny Suryadi, Daniel Chiung-Jui Su, Wei-Chuan Wang, Wai Wah Lai, Yuanita Ida, Kentaro Onishi

This supplementary file contains the following supporting information cited in the main manuscript:

- Table S1 — Individual raw WOMAC domain and total scores at baseline and follow-up.
- Table S2 — Exploratory inferential analyses (Friedman and Wilcoxon signed-rank tests).
- Table S3 — Descriptive follow-up context (BMI, cointerventions, additional interventions).
- Video S1 — Dynamic procedural demonstration of the ultrasound-guided posteromedial fascial-periarticular injection approach.

Table S1. Individual raw WOMAC domain and total scores at baseline and follow-up.

Patient	Baseline Pain	Baseline Stiff.	Baseline Func.	Baseline Total	1-Month Pain	1-Month Stiff.	1-Month Func.	1-Month Total	3-Month Pain	3-Month Stiff.	3-Month Func.	3-Month Total	6-Month Pain	6-Month Stiff.	6-Month Func.	6-Month Total	12-Month Pain	12-Month Stiff.	12-Month Func.	12-Month Total
P001	13	5	42	60	9	3	28	40	8	4	24	36	6	2	22	30	5	2	23	30
P002	14	5	45	64	9	4	30	43	8	3	28	39	7	2	26	35	6	2	23	31
P003	10	4	38	52	6	2	22	30	5	2	20	27	5	2	19	26	5	2	17	24
P004	13	5	44	62	8	4	30	42	7	3	27	37	6	3	23	32	6	2	22	30
P005	12	4	40	56	7	3	25	35	6	3	21	30	6	2	21	29	5	2	19	26
P006	14	5	44	63	9	4	30	43	8	3	28	39	7	3	24	34	7	2	22	31
P007	12	5	42	59	7	3	27	37	6	2	23	31	6	2	21	29	6	2	19	27
P008	13	5	42	60	8	3	28	39	7	3	25	35	6	2	22	30	5	2	23	30
P009	11	4	39	54	6	3	25	34	6	2	22	30	5	2	20	27	5	2	19	26
P010	14	5	46	65	9	4	30	43	8	3	28	39	7	3	24	34	6	3	21	30
**Mean**	**12.6**	**4.7**	**42.2**	**59.5**	**7.8**	**3.3**	**27.5**	**38.6**	**6.9**	**2.8**	**24.6**	**34.3**	**6.1**	**2.3**	**22.2**	**30.6**	**5.6**	**2.1**	**20.8**	**28.5**

Abbreviations: WOMAC, Western Ontario and McMaster Universities Osteoarthritis Index. Higher scores indicate worse symptoms and disability. Raw patient-level scores were recorded at baseline before treatment and at 1, 3, 6, and 12 months after the final injection. Cell values verified against Table 2 during the Major Revision (all column means match Table 2 exactly).

Table S2 — Exploratory Inferential Analyses (new)

Table S2. Exploratory inferential analyses of WOMAC scores across follow-up (n = 10).

Domain	Friedman $\chi^2(4)$	Friedman p	Wilcoxon vs Baseline raw p	Wilcoxon Bonferroni p (k = 4)
Pain	37.97	1.14×10^{-7}	1.95×10^{-3} (all timepoints)	7.81×10^{-3}
Stiffness	34.87	4.94×10^{-7}	1.95×10^{-3} (all timepoints)	7.81×10^{-3}
Function	38.45	9.04×10^{-8}	1.95×10^{-3} (all timepoints)	7.81×10^{-3}
Total	39.68	5.05×10^{-8}	1.95×10^{-3} (all timepoints)	7.81×10^{-3}

Wilcoxon p reaches the theoretical minimum at n = 10 ($2^{-9} = 1.95 \times 10^{-3}$) because all within-patient changes were in the same (improvement) direction at every timepoint. Bonferroni correction was applied for k = 4 pairwise comparisons per domain. Paired t-tests and Cohen's dz for WOMAC total are provided in Table 3 of the main manuscript. All inferential analyses are exploratory and hypothesis-generating; the small sample size (n = 10) does not permit confirmatory causal inference. Analyses were performed in Python 3 (SciPy).

Table S3 — Descriptive Follow-up Context (new)

Table S3. Descriptive follow-up context — BMI, cointerventions, and additional interventions during the 12-month follow-up period (n = 10).

Variable	Baseline	12 months	Notes
BMI (kg/m²), mean ± SD	26.4 ± 2.8	26.3 ± 2.7	Δ < 0.5 kg/m² across cohort
Ongoing physiotherapy, n (%)	10 (100%) prior to injection	3 (30%) intermittent during FU	Not intensified vs baseline
Regular oral analgesic/NSAID use, n (%)	10 (100%)	2 (20%) as-needed only	Substantially reduced
Additional intra-articular knee injection, n (%)	—	0 (0%)	None
Additional peri-articular knee injection, n (%)	—	0 (0%)	None
Knee surgery during follow-up, n (%)	—	0 (0%)	None
Lifestyle/exercise change, n (%)	—	4 (40%) reported increased activity	Descriptive only
Any additional systemic pharmacotherapy for OA, n (%)	—	0 (0%)	None
Protocol deviation, n (%)	—	0 (0%)	All 10 patients received 3 planned injections
Loss to follow-up, n (%)	—	0 (0%)	Complete 12-month WOMAC data

Data extracted from routine clinical registry notes rather than a structured trial case report form; reported descriptively as contextual variables only, not as adjusted covariates. Abbreviations: BMI, body mass index; FU, follow-up; NSAID, non-steroidal anti-inflammatory drug; OA, osteoarthritis; SD, standard deviation.

Video S1 legend

Video S1. Dynamic procedural demonstration of the ultrasound-guided posteromedial fascial-periarticular injection approach.

The video demonstrates patient positioning, transducer placement, ultrasound-based identification of the posteromedial target region, and in-plane needle advancement for injectate delivery. Representative still frames from this video are shown in Figure 1 of the main manuscript. Higher-resolution playback is recommended to clearly appreciate the sonoanatomic labeling.

URL: https://www.dropbox.com/scl/fi/1xt8b8f6ign9rmp470lr6/PF-injection-still-image-with-labelling.mp4?rlkey=ed3cfj8icf2hrn8fjfqrifj9i&dl=0

Disclaimer/Publisher's Note: *The statements, opinions and data contained in all publications are solely those of the individual author(s) and contributor(s) and not of MDPI and/or the editor(s). MDPI and/or the editor(s) disclaim responsibility for any injury to people or property resulting from any ideas, methods, instructions or products referred to in the content.*
